# Functional Analysis of the Gonococcal Genetic Island of *Neisseria gonorrhoeae*

**DOI:** 10.1371/journal.pone.0109613

**Published:** 2014-10-23

**Authors:** Emilia Pachulec, Katja Siewering, Tobias Bender, Eva-Maria Heller, Wilmara Salgado-Pabon, Shelly K. Schmoller, Katelynn L. Woodhams, Joseph P. Dillard, Chris van der Does

**Affiliations:** 1 Department of Microbiology, Groningen Biomolecular Sciences and Biotechnology Institute, University of Groningen, Groningen, The Netherlands; 2 Department of Ecophysiology, Max-Planck-Institute for Terrestrial Microbiology, Marburg, Germany; 3 Department of Medical Microbiology and Immunology, University of Wisconsin, Madison, Wisconsin, United States of America; University of Arkansas for Medical Sciences, United States of America

## Abstract

*Neisseria gonorrhoeae* is an obligate human pathogen that is responsible for the sexually-transmitted disease gonorrhea. *N. gonorrhoeae* encodes a T4SS within the Gonococcal Genetic Island (GGI), which secretes ssDNA directly into the external milieu. Type IV secretion systems (T4SSs) play a role in horizontal gene transfer and delivery of effector molecules into target cells. We demonstrate that GGI-like T4SSs are present in other β-proteobacteria, as well as in α- and γ-proteobacteria. Sequence comparison of GGI-like T4SSs reveals that the GGI-like T4SSs form a highly conserved unit that can be found located both on chromosomes and on plasmids. To better understand the mechanism of DNA secretion by *N. gonorrhoeae*, we performed mutagenesis of all genes encoded within the GGI, and studied the effects of these mutations on DNA secretion. We show that genes required for DNA secretion are encoded within the *yaa-atlA* and *parA-parB* regions, while genes encoded in the *yfeB-exp1* region could be deleted without any effect on DNA secretion. Genes essential for DNA secretion are encoded within at least four different operons.

## Introduction

Type IV secretion systems (T4SSs) are large multiprotein complexes used by many bacteria to transport macromolecular substrates across membranes (for reviews see [1]–[6]). The T4SS of *N. gonorrhoeae* is located on a horizontally acquired 57 kb variable genetic island (the Gonococcal Genetic Island, GGI) [7], [8]. The GGI is present in approximately 80% of the *N. gonorrhoeae* strains and 17% *N. meningitidis* strains [7], [9], [10]. The T4SS in *N. gonorrhoeae* strain MS11 secretes ssDNA into the environment [7], [8]. The secreted DNA is used for natural transformation of other *N. gonorrhoeae* cells and therefore contributes to horizontal gene transfer [7], [8], [11] and was shown to facilitate especially the initial phases of biofilm formation in continuous flow-chamber systems [12]. The secretion of DNA occurs during the log-phase and is higher in strains expressing Type IV pili than in strains which do not express Type IV pili [13]. Secretion seems to be regulated at the transcription level of the operon encoding the coupling protein and the relaxase [13], [14]. The role of the GGI in pathogenesis is currently still unclear. Different forms of the GGI have been identified, and some of these forms are preferentially found in disseminated gonococcal infection isolates [7]. During intracellular infection, the presence of the T4SS allows for survival of *N. gonorrhoeae* strains lacking the Ton complex, which is otherwise required for intracellular growth due to its role in the uptake of iron [15]. Remarkably, the T4SS present in *N. meningitidis* does not secrete DNA, nor does it confer Ton-independent intracellular survival [10].

**Figure 1:**
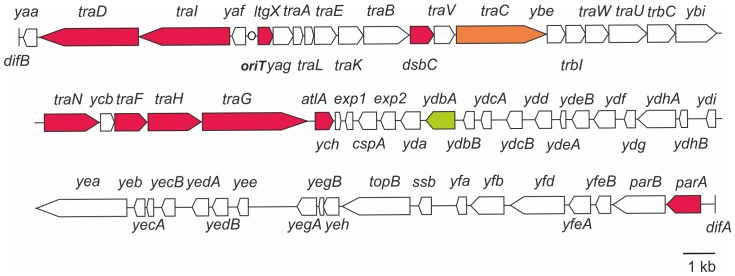
Schematic representation of the genetic map of the GGI of *N. gonorrhoeae*. Genes that have been previously characterized are colored. Red and orange indicate genes in which mutations resulted in a decrease in DNA secretion. Green indicates genes in which mutations had no effect on DNA secretion. For the genes colored in red, secretion could be restored by complementation. For the genes colored in orange no complementation was performed. The dot indicates the origin of transfer (*oriT*). difA and difB indicate the respective dif sites flanking the GGI.

The GGI of *N. gonorrhoeae* strain MS11 encodes at least 63 putative open reading frames (Figure 1). Within the first 27.5 kb of the GGI, 27 ORFs are encoded, 22 of which encode for proteins (Yaa, TraD, TraI, LtgX, TraA, TraL, TraE, TraK, TraB, DsbC, TraV, TraC, TrbI, TraW, TraU, TrbC, Ybi, TraN, TraF, TraH, TraG, and AtlA) that show significant similarity to proteins of other T4SSs mainly the F plasmid T4SS [8]. For 5 proteins (Yaf, Yag, Ybe, Ycb and Ych) no homologs of known function could be identified. Genes in the first 27.5 kb are transcribed in two different directions, demonstrating the presence of at least two, but possibly more operons. Between these two putative operons the origin of transfer (*oriT*) is located [16]. The second 29.5 kb, from *exp1* to the other *dif* site encodes at least 36 ORFs. Several of the proteins within this region encode proteins with homology to DNA processing and modifying proteins, however most genes encode for proteins with an unknown function [8]. Previous mutational analysis identified several genes in the genetic island (*traD*, *traI*, *ltgX*, *dsbC*, *traC*, *traN*, *traF*, *traH*, *traG*, *atlA* and *parA*) essential for DNA secretion [8], [17]. Mutagenesis of *ydbA* was the only currently-identified mutation that did not affect DNA secretion (Figure 1). In this study we show that GGI-like T4SSs are present in several other bacteria. We then set out to create mutations in all previously uncharacterized genes of the GGI and tested these mutants in DNA secretion assays. Finally, we determined the operon structures of genes involved in this process.

## Materials and Methods

### Bacterial strains and plasmids

Bacterial strains and plasmids used in this study are described in Table S1 and Table S2 respectively. Mutagenesis studies were performed using *N. gonorrhoeae* MS11. Gonococci were grown on GCB (Difco) plates containing Kellogg's supplements [18] or in GCBL liquid medium (GCBL) containing 0.042% NaHCO_3_ [19] and Kellogg's supplements. For blue/white screening, *N. gonorrhoeae* was grown on GCB-Tris-XP plates [20]. *E. coli* strains were grown on Luria-Bertani (LB) broth or on LB agar plates. Chloramphenicol was used at 10 µg/ml, and erythromycin at 10 µg/ml and 500 µg/ml for *N. gonorrhoeae* and *E. coli* respectively.

### Construction of gonoccocal mutants

Gonococcal mutants were created using different techniques. Insertion-duplication mutagenesis [17] was used to generate polar and effectively ‘nonpolar’ mutants. In brief, an internal part of the gene of interest was amplified using PCR primers described in Table S3. This PCR product was cloned into either the pIDN1 or pIDN3 plasmids [17]. The generated plasmids are described in Table S2. These plasmids were then transformed to *N. gonorrhoeae* MS11 by natural transformation [21]. Transformants were selected on erythromycin, and correct insertion was determined by PCR analysis, and confirmed by sequencing of the PCR products. The pIDN1 and pIDN3 plasmids contain an *ermC* cassette with a strong promoter. If the targeted gene is inserted in a similar orientation as the *ermC* cassette, downstream genes will, after insertion of the plasmid into the chromosome via single crossover, still be transcribed. This method using the pIDN1 and pIDN3 plasmids was shown to create insertions that are effectively nonpolar on the transcription of downstream genes [8], [13], [16], [17]. Using this strategy, ‘nonpolar’ mutants in *traK*, *traE*, *traW*, *traU*, *trbC* and *parB* were created. By inserting the targeted gene in the opposite direction as the *ermC* cassette a polar insertion can be created. Such polar mutants were created in *yea* and *topB*. Alternatively, instead of inserting the entire pIDN1 or pIDN3 vector into the chromosome, only the *ermC* cassette can be integrated into the chromosome via double recombination to replace the targeted gene. Similarly, this will result in deletions that are effectively nonpolar on the transcription of downstream genes. To facilitate such an approach, pSH001, a vector with multiple cloning sites upstream and downstream of the *ermC* cassette was constructed. To construct pSH001, two PCR products amplified using pIDN1 as template with primers ForwardDUS and ReverseDUS and primers ForwardErmC and ReverseErmC respectively, were digested with EcoRI and HindIII and ligated. To create mutants using this vector, flanking regions of the gene of interest were amplified using PCR primers described in Table S3, and cloned in the multiple cloning sites upstream and downstream of the *ermC* cassette of pSH001. These plasmids were then transformed to *N. gonorrhoeae* MS11 by natural transformation, and transformants were selected on erythromycin. The presence of the insertion was determined by PCR, and confirmed by sequencing of the PCR products. ‘Nonpolar’ mutants of *traB*, *traC*, *traV* and *traL* were obtained using this approach. Markerless mutations can be created by transformation with a plasmid or PCR product containing the up and downstream regions of the gene, but not the gene itself. Mutation of *traA*, *trbI* and *ybe* were generated cloning the upstream and downstream regions of the genes into pIDN1. Transformation of these plasmids into MS11 followed by double homologous recombination resulted in in-frame deletions of these genes. The presence of the deletion was determined by PCR, and confirmed by sequencing of the PCR products. To create a markerless in-frame deletion of *yaf*, the flanking regions of *yaf* were amplified with primers 41 and 812R-GGI, and primers 808R-GGI and 813F-GGI. The products were digested with EcoRI and ligated. The ligation product was amplified by PCR using primers 41 and 808R-GGI, and transformed to *N. gonorrhoeae* MS11, resulting in strain TB001. A deletion of *ycb* was made in pKS68 by PCR with primers ycbBsaF and ycbBsaR. The PCR product was digested with BsaI and ligated to produce pSI11 containing a 387 bp in-frame deletion in *ycb*, removing 91% of the *ycb* coding region. The deletion was introduced into *N. gonorrhoeae* MS11 by natural transformation without selection, and the presence of the deletion was determined by PCR. The *ycb* deletion mutant was designated SI11. Similarly, a deletion of *yag* was made in pKS96 by PCR with primers yag3′F and yag5′R, digestion with BsaI, and ligation to generate pSI10. The deletion removed the entire coding region between the start and stop codon. The deletion was introduced into *N. gonorrhoeae* MS11 by natural transformation without selection, and the *yag* deletion mutant was designated SI10. An in-frame deletion in *ybi* was created by digestion of pKS78 with PstI, blunting with T4 DNA polymerase and ligation to generate pSI12. The deletion removed 53% of the *ybi* coding region near the 5′ end of the gene. The deletion was introduced into *N. gonorrhoeae* MS11 by natural transformation without selection and the *ybi* deletion mutant was designated SI12. An insertion in *ych* was made by shuttle mutagenesis of pJD1103 with mTn*CmNS* generating pJD1188. The location of the insertion was determined by DNA sequencing. The insertion was found 68 bp from the translational start. Thus the truncated protein would maintain 44% of the N-terminal coding region. The insertion was introduced into *N. gonorrhoeae* strain MS11 by natural transformation, and transformants were selected using chloramphenicol, resulting in strain JD1614.

The *yaa* region was amplified from strain MS11 and ligated into pIDN1, generating pKL8. A frame-shift mutation was introduced into *yaa* by digestion of pKL8 with AgeI followed by treatment with T4 DNA polymerase, creating a 4 bp insertion followed immediately by a stop codon resulting in plasmid pKL9. *N. gonorrhoeae* strain MS11 was transformed with pKL9 without selection, and potential transformants were screened by PCR of the *yaa* region followed by digestion of the PCR product with AgeI and EagI. One such transformant, KL505, was found to have incorporated the *yaa* mutation thus encoding a Yaa protein truncated at amino acid 50.

A *yag* complementation construct was made by PCR amplifying *yag* from MS11 chromosomal DNA using primers yagRBSF and 48R. The resulting product was digested with HindIII and SpeI and ligated into similarly digested pKH37, generating pPK1007. The complementation construct was introduced into the *N. gonorrhoeae yag* mutant SI10 chromosome by natural transformation. Transformants were selected with chloramphenicol and screened by PCR for the presence of *yag* at the complementation site between *aspC* and *lctP* and for the maintenance of the *yag* deletion at the native locus. The *yag* complemented strain was designated PK153. A *parB* complementation construct was made by PCR amplifying *parB* from MS11 chromosomal DNA using primers GGI-224F and GGI-225R. The resulting product was digested with SalI and SacI and ligated into similarly digested pKH35, generating pEP056. The complementation construct was introduced into the *N. gonorrhoeae parB* mutant EP013 by natural transformation. Transformants were selected with chloramphenicol and screened by PCR for the presence of *parB* at the complementation site between *aspC* and *lctP*. The *parB* complemented strain was designated EP046. To restore the *yaa*-locus in *N. gonorrhoeae* strain KL505 *yaa* was amplified from *N. gonorrhoeae* MS11 chromosomal DNA with primers 832F-GGI and 833R-GGI. The resulting PCR product was transformed into *N. gonorrhoeae* KL505, resulting in strain TB003. Deletion of the *exp1-yfeB* region of the GGI was created by transformation of *N. gonorrhoeae* strain HH522 [8] with construct pEP022. pEP022 was created as follows. The 1081 bp region of *atlA-exp1* was amplified with primers GGI-105F and GGI-106R. The resulting product was digested with SalI and ApaI and cloned into pIDN2, resulting in pEP021. Then, the *parA*-*parB* region was amplified with primers GGI-107F and GGI-108R, digested with EcoRI and NotI and ligated into pEP021, resulting in pEP022. This plasmid was then transformed to *N. gonorrhoeae* HH522 resulting in strain EP016. An isolate in which the plasmid was integrated into the chromosome was selected on erythromycin and screened on XP plates for white colonies.

### Synteny analysis

To identify GGI-like T4SSs in other bacteria, all GGI encoded proteins were screened for synteny using the absynte webtool (http://archaea.u-psud.fr/absynte/). Using this approach, several GGI-like T4SSs could be identified in other Proteobacteria. The genetic regions encoding proteins homologous to components of the T4SS of *N. gonorrhoeae* were visually analyzed with the Microbial Genome Viewer 2.0 (http://mgv2.cmbi.ru.nl/genome/index.html).

PSI-Blast (http://www.ncbi.nlm.nih.gov/blast/Blast.cgi) was used to identify homologues of all genes surrounding the GGI-like regions, either for proteins encoded within the GGI or for other proteins.

### Transcriptional Mapping

Piliated *N. gonorrhoeae* strains were transferred to new plates until non-piliated colonies appeared. These non-piliated colonies were further transferred to obtain plates with non-piliated colonies. Cells obtained from plates were transferred to tubes with GCBL liquid medium containing 0.042% NaHCO_3_ and Kellogg's supplements and were grown until OD_600_∼0.6 was reached. Total RNA of 1 ml culture was isolated using the peqGOLD TriFast reagent (PeqLab). To remove contaminating DNA, total RNA was treated with 1 unit RNase-free DNaseI (Fermentas) for 30 min at 37°C. RNA was quantified spectrophotometrically, and quality assessed by agarose gel electrophoresis. The MuLV transcriptase and the random hexamer primer of the first strand cDNA synthesis kit (Fermentas) were used to generate cDNA. A control of cDNA synthesis was performed without MuLV transcriptase. Transcripts were mapped using the primers described in Table S3.

### Quantitative PCR

Transcript levels of *traI*, *traD*, *ltgX*, *traH* and *parA* and the reference gene *secY* were determined for RNA isolated from MS11, two *Δyaa* strains (KL505 and pTB002), and the KL505 derivative in which the reading frame of *yaa* was restored (TB003) by quantitative Real-Time PCR (qRT-PCR). Oligonucleotide primers were designed using Clone Manager 9 professional edition (Sci-Ed Software). The primers used are described in Table S3. cDNA was synthesized as described above for transcriptional mapping. qRT-PCRs were performed using the SYBR Green/ROX qPCR Master Mix (Fermentas) in a 7300 Real Time PCR System of Applied Biosystems. Reaction mixtures were prepared in a 25 µl volume and run in triplicate for each gene. Chromosomal DNA of *N. gonorrhoeae* strain MS11 was used to establish the primer efficiency. Three biological replicates were performed. Results were depicted as the level of transcript compared with the *secY* gene (2^∧−ΔCt^).

### DNA secretion assay

Non-piliated colonies were identified, streaked on GCB agar plates, and grown overnight. Colonies from agar plates were then inoculated in 3 ml of Graver Wade medium [22]. These cultures were grown for 1.5 h and then diluted to OD_600_∼0.2. To reduce the DNA background from lysed cells, cultures were grown twice more for 2 hours, and each time diluted again to OD_600_∼0.1. From the last round of growth samples were collected directly after dilution, and after 2 hours. Experiments were performed for 6 independent cultures, and repeated at least twice. Supernatants were assayed for the amount of DNA using PicoGreen (Molecular Probes), which can bind both dsDNA and ssDNA. Tecan software was applied for fluorescence measurement of culture supernatants. The amount of secreted DNA was calculated from an ssDNA standard curve divided by the increase of the OD_600_. In all experiments at least strain MS11 (WT) and ND500 (MS11ΔGGI) were included. The fluorescence value obtained for ND500 was substracted as background.

## Results and Discussion

### GGI-like T4SSs are found in several β-and γ-Proteobacteria

**Figure 2:**
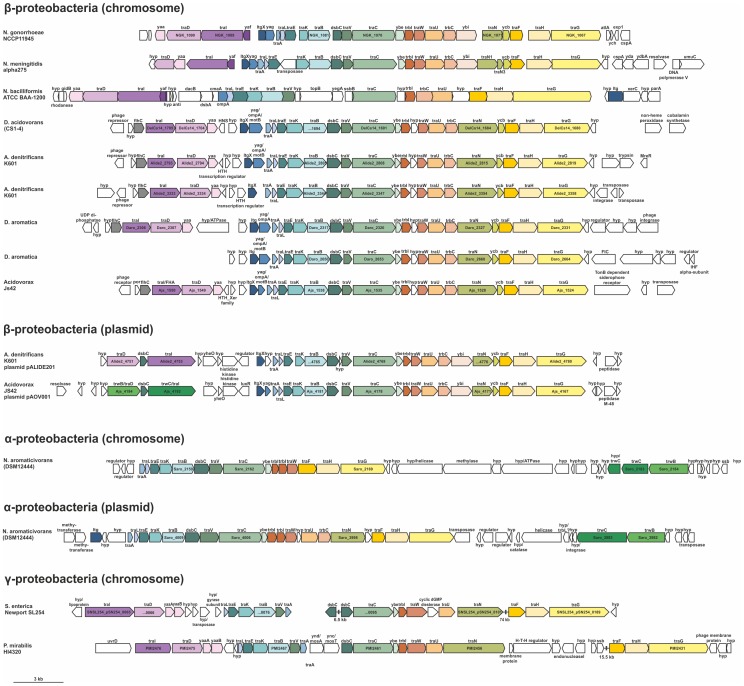
Genetic organization of genome and plasmid encoded GGI-like T4SSs identified in α-, β-, and γ-proteobacteria. The official numbering and nomenclature of the genes is indicated within the gene-arrows. The corresponding homologues are indicated on top of the arrows. T4SS genes are indicated by coloring, homologous genes are labeled in equivalent colors. The lengths of the arrows correspond to the lengths of the annotated genes. A scale bar of 3 kb is given on the right bottom of the schematic.

Different variants of the GGI have been identified in *N. gonorrhoeae*, and *N. meningitidis* [7], [9], [10], [23], and the GGI has been also found in a whole genome shotgun sequence of *Neisseria bacilliformis* (GenBank: AFAY01000002.1). To determine whether GGI-like T4SSs are present in other bacteria or are specific to the order of the *Neisseriaceae*, all proteins encoded within the GGI were screened for synteny. Using this approach, several GGI-like T4SSs could be identified in other proteobacteria (Figure 2). Most of the organisms containing a GGI-like T4SSs belong to the group of β-proteobacteria, but GGI-like T4SSs were also identified in the pathogenic γ-proteobacteria *Salmonella enterica* and *Proteus mirabilis*, and in the α-proteobacterium *Novosphingobium aromaticivorans*. Most of these T4SSs are located on the chromosome, but also T4SSs were identified on the plasmids of *Alcaligenes denitrificans*, *Acidovorax* JS42 and *N. aromaticivorans*. Interestingly, the three strains that contained a plasmid encoding a GGI-like T4SS also encoded a GGI-like T4SS on the chromosome. Most of the T4SSs seemed to encode a full set of T4SS proteins with homology to the T4SS encoded in the GGI, which were encoded in a similar order as observed for the GGI. Analysis of the regions flanking the GGI-like T4SSs often showed the presence of integrases, resolvases and transposases typically found in mobile genetic elements. However, no clear conservation of the flanking regions could be detected. The *parA-exp1* region is only found associated with the GGI-like T4SS of *Neisseriaceae*. Furthermore, although the gonococcal and meningococcal GGIs are inserted at the *dif* site [8], [10], [24] no specific site of insertion into the chromosome could be determined for other GGI-like sequences. Taken together, the organization of the genes encoding the T4SS proteins, but not the surrounding regions, highly conserved among GGI-like T4SS.

### Analysis of the genes encoded in the yaf-yaa region of the GGI

A more detailed analysis of the GGI-like T4SSs shows that most GGI-like T4SSs encode homologs of the relaxase TraI, the coupling protein TraD and the small, putative inner membrane protein Yaa (Figure 2). These relaxases are mostly members of the MOB_H_ family of relaxases, which are often associated with a TraD-like coupling protein and a small, putative inner membrane protein. Only the GGI-like T4SS encoded on the pAOV001 plasmid of *Acidovorax* JS42 and the chromosomal and plasmid encoded GGI-like T4SSs of *N. aromaticivorans* encode a relaxase of the MOB_F_ family of relaxases, which are often linked to T4SS transport complexes of the MPF_F_ family. Indeed, the transport complexes of the GGI-like T4SSs belong to the MPF_F_ family. Thus the transport complexes of GGI-like T4SSs might function both with relaxases of the MOB_H_ and the MOB_F_ family. Indeed different combinations of targeting (MOB) and transport (MPF) classes have been previously observed [25]–[27]. No relaxase could be identified for one of the two GGI-like T4SSs encoded on the chromosome of *D. aromatica*, suggesting that both systems might use the same relaxase. Homologs of the small, putative inner membrane protein Yaa are always found downstream of the coupling protein encoded in GGI-like T4SSs which contain a MOB_H_ relaxase. The *yaf* gene located upstream of the *traI* gene was only found in the GGI-like T4SSs of *N. meninigitidis* and *N. bacilliformis*. The protein encoded in the *yaf* gene has no homology with other proteins. Based on its position and its size, *yaf* might encode a regulatory or a nicking accessory protein. Interestingly, several other GGI-like T4SSs encode homologs of the flagellar transcriptional activator (FlhC) at this position.

**Figure 3:**
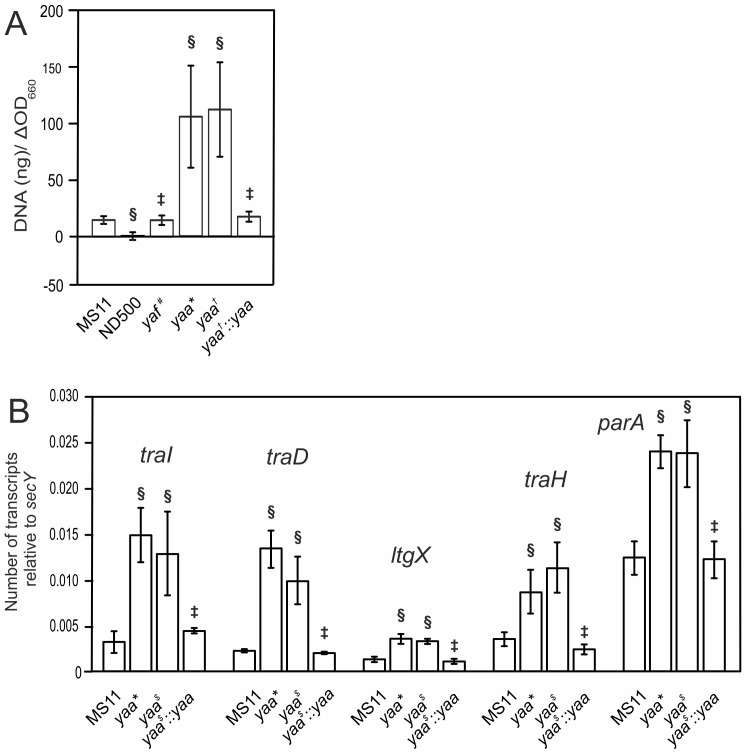
Characterization of the *yaf-yaa* region. A) Fluorometric detection of secreted DNA of mutants created in the *yaf-yaa* region Gonococcal strains were repeatedly diluted in liquid culture. Cell-free culture supernatants were collected, and DNA was detected with the fluorescent DNA-binding dye PicoGreen and normalized to the increase in the OD_600_. MS11 was used as the wild-type (WT) strain and ND500 (MS11ΔGGI) was used for the background value. *yaf^#^* indicates the TB001 strain which contains a markerless in-frame deletion of *yaf*. yaa* indicates the TB002 strain in which the *yaa* gene is disrupted by insertion of an *ermC* containing plasmid via insertion duplication mutagenesis. *yaa^†^* indicates the KL505 strain in which a 4 bp insertion creates a frame shift in *yaa*, and *yaa^†^::yaa* indicates the TB003 strain in which the 4 bp insertion is removed via homologous recombination with WT *yaa*. B) Quantitative gene expression levels of the *traI*, *traD*, *ltgX*, *traH* and *parA* genes of non-piliated *N. gonorrhoeae* strains were determined by qRT-PCR. The graph shows the mRNA levels as comparative gene expression after normalizing each gene to *secY*. Values depict means ± standard deviation of at least six biological replicates. § indicates a Student's T-test P-value≤0.05 compared to wild-type; ‡, indicates no statistical difference compared to WT.

Previously it was shown that TraI and TraD encoded within the GGI are essential for DNA secretion [8], [16], [28] (Figure 1), however the role of *yaf* and *yaa* in DNA secretion has not been characterized. In order to evaluate the role of *yaf* and *yaa* in DNA secretion, a markerless in-frame deletion of *yaf* was created, and the *yaa* gene was disrupted by insertion of an *ermC* containing plasmid via insertion-duplication mutagenesis. Deletion of *yaf* did not have any effect on DNA secretion (Figure 3A) demonstrating that *yaf* is not essential for DNA secretion. Remarkably, the amount of DNA detected in the medium of the *yaa* insertion-duplication mutant strain was highly variable and was approximately 7-fold higher than the amount of DNA secreted by the WT MS11 strain (Figure 3A). To confirm that the increased DNA levels were due to the *yaa* disruption, and were not an effect of the insertion of the *ermC* containing plasmid or a secondary mutation, a second *yaa* disruption strain was created. The newly created *yaa* mutant had a 4-base insertion within *yaa* resulting in a frame-shift mutation. Again DNA levels in the medium were highly variable, and an average 7-fold increase in the amount of DNA was observed (Figure 3A). To further confirm these results, a strain was created in which the 4-base insertion in the *yaa* gene was repaired. DNA levels in the medium for this strain were similar to the levels observed for the WT MS11 strain (Figure 3A). Thus disruption of *yaa*, either by chromosomal insertion or by introduction of a frame-shift mutation, resulted in higher levels of DNA in the medium. It has previously been demonstrated that DNA secretion seems to be regulated at the transcriptional level of the putative operon containing *traI* and *traD* [28]. To test whether the observed increase in DNA in the medium of *yaa* mutants was due to an increase in the transcription levels of T4SS components, the expression of *traI*, *traD*, *ltgX*, *traH* and *parA* genes was determined by real-time RT-PCR. The expression levels of these genes were normalized to the expression level of *secY*, the constitutively-expressed, large membrane component of the SecYEG complex involved in protein translocation. The transcription of *traI*, *traD*, *ltgX*, *traH* and *parA* were increased in both *yaa* mutants. Interestingly, the transcription of the *traI* and *traD* genes was more upregulated than the transcription of the *ltgX*, *traH* and *parA* genes. The expression levels of *traI*, *traD*, *ltgX*, *traH* and *parA* in the strain with the restored *yaa-*locus were similar to the levels observed in the WT MS11 strain (Figure 3B). Taken together, these results show that in both *yaa* mutants the transcription of the genes of the T4SS are increased, which might lead to the increased release of DNA was observed. The only Yaa homolog that has been further studied is the s043 protein, which is the Yaa homolog of the *Vibrio cholerae* SXT integrative and conjugative element. Mutagenesis of *s043* resulted in a strain unable to conjugate [29]. Thus mutations in the genes encoding the relaxase TraI, the coupling protein TraD, and the small conserved membrane protein Yaa, affect the DNA levels in the medium, whereas mutagenesis of *yaf* has no effects. The exact mechanism by which mutagenesis of *yaa* results in increased levels of DNA in the medium remains unknown.

### Analysis of the genes encoded in the ltgX-ych region of the GGI

**Figure 4:**
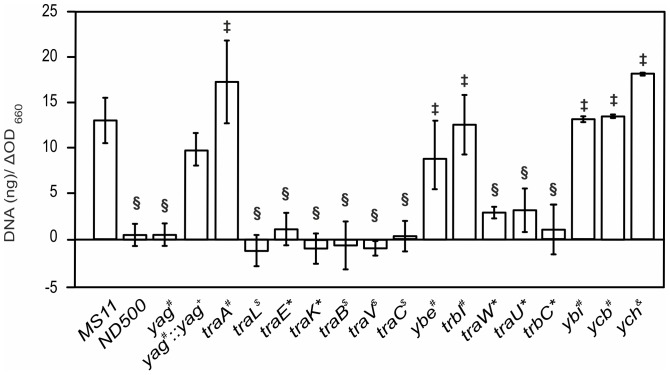
Characterization of the *ltgX-atlA* region. A) Fluorometric detection of secreted DNA of mutants created in the *ltgX-atlA* region was performed as in Figure 2. # indicates a strain which contains a markerless in-frame deletion of the respective gene. * indicates a strain in which the respective gene is disrupted by insertion of an *ermC* containing plasmid via insertion duplication mutagenesis. $ indicates a strain in which the gene is disrupted by replacement with the *ermC* cassette via double homologous recombination. *yag^#^*::*yag^+^* indicates the complementation mutant of the in-frame deletion of *yag* by expressing *yag* from the complementation site between the *aspC* and *lctP* genes. *ych^&^* indicates the strain in which *ych* is disrupted by a transposon insertion. § indicates a Student's T-test P-value≤0.05 compared to wild-type; ‡, indicates no statistical difference compared to WT.

A comparison of the *ltgX-ych* region in which the components of the transport complex and pilus assembly are encoded shows that the genes encoding the TraA, TraL, TraE, TraK, TraB, DsbC, TraV, TraC, Ybe, TrbI, TraW, TraU, TrbC, TraF and TraH proteins are conserved in almost all of the identified GGI-like T4SSs. Genes encoding homologs of LtgX, Yag, Ybi, TrbC, Ycb and Ych are missing from several of the GGI-like T4SSs. Remarkably, no homologs of the lytic transglycosylase AtlA were identified in other GGI-like T4SSs, but AtlA is essential for DNA secretion in *N. gonorrhoeae* [17], [23], [30], which indicates that it has a special function within the *N. gonorrhoe*ae T4SS. Homologs of the TraN and TraG inner membrane proteins are found in all GGI-like T4SSs, but the predicted size of these proteins differs strongly between strains. Several GGI-like T4SSs encode a small hypothetical protein not found in the GGI between the *trbI* and *traW* genes. Homologs of the pilin protein TraA and the pilin circularizing protein TrbI are found in all GGI-like T4SSs. All *traA* genes encode full length TraA proteins, suggesting that these T4SSs are able to synthesize a circular pilin subunit. Several mutants previously created in this region (*ltgX*, *traH*, *traF*, *traN*, *traC*, *dsbC*, *traG* and *atlA*) affected DNA secretion [8], [17], [30] (Figure 1). To complete the previously published analysis, the remaining genes were mutated either by insertion-duplication mutagenesis or by creating a complete deletion. Analysis of the new mutants revealed that mutation of the *yag*, *traL*, *traE*, *traK*, *traB*, *traV*, *traC*, *traW*, *traU* and *trbC* genes abolished DNA secretion, while mutagenesis of the *traA*, *ybe*, *trbI*, *ybi*, *ycb* and *ych* genes did not influence secretion (Figure 4).

Interestingly, mutagenesis of *yag*, a gene which is found in several GGI-like T4SS, but that has no homologs in other T4SS, resulted in a strong reduction of DNA secretion. To exclude any polar effects of the *yag* deletion, the markerless in-frame deletion of *yag* was complemented by expressing *yag* from the complementation site between *aspC* and *lctP*. Indeed complementation of the *yag* mutant resulted in normal levels of DNA secretion (Figure 4), demonstrating that Yag is important for DNA secretion. Yag is a small (173 amino acid) protein with a putative signal peptide and an OmpA-like domain. OmpA-like domains have been shown to non-covalently associate with peptidoglycan [31]. Thus, Yag is most likely secreted to the periplasm, where it associates with the peptidoglycan layer. However, the function of the Yag protein is currently still unknown.

Mutagenesis of the genes encoding the TraA pilin and TrbI, the enzyme that circularizes the full length TraA pilin did not affect DNA secretion. The *traA* gene of *N. gonorrhoeae* strain MS11 contains a frameshift mutation, which results in a truncation of the last 14 amino acids and expression of a pilin subunit that cannot be circularized [32]. Thus it is not surprising that mutagenesis of the *trbI* gene, in a strain containing a defective *traA* gene, does not affect DNA secretion. The presence of a functional pilin thus is not required for DNA secretion in *N. gonorrhoeae*, which further confirms that pilus assembly and substrate transport by T4SSs are two separated processes. Substrate transport in the absence of a detectable conjugative pilus has previously been reported for the *A. tumefaciens* T4SS [33]–[36] and the *B. pertussis ptl* system [37]. Ybe is a small hypothetical protein with one predicted transmembrane domain, which is found in almost all GGI-like T4SS. No homologs of Ybe are found in other T4SSs like *e.g.*, the F plasmid. The fact that Ybe is encoded upstream of TrbI and is not involved in DNA secretion might indicate a role in pilin processing or assembly.

Ycb and Ybi are missing from several of the GGI-like T4SSs, and no homologs of Ych were identified in other T4SSs. Ybi has some similarity near the C-terminus to part of TraN proteins from some conjugation systems [8]. Mutagenesis of *ybi*, *ycb* and *ych* did not have an effect on DNA secretion, demonstrating that the proteins encoded by these genes do not play a role in DNA secretion (Figure 4). Taken together, most of the genes in the *ltgX-ych* region, that are conserved in GGI-like T4SSs (*traL*, *traE*, *traK*, *traB*, *dsbC*, *traV*, *traC*, *traW*, *traU*, *trbC*, *traN*, *traF* and *traH*) and three genes (*ltgX*, *trbC* and *yag*) that are found in most, but not all GGI-like T4SS are important for DNA secretion. Only mutations in *traA*, *trbI*, *ybe*, *ybi*, *ycb* and *ych*, do not affect DNA secretion via the T4SS encoded within the GGI.

### parA and parB, but not the exp1-yfeB region are required for DNA secretion

**Figure 5:**
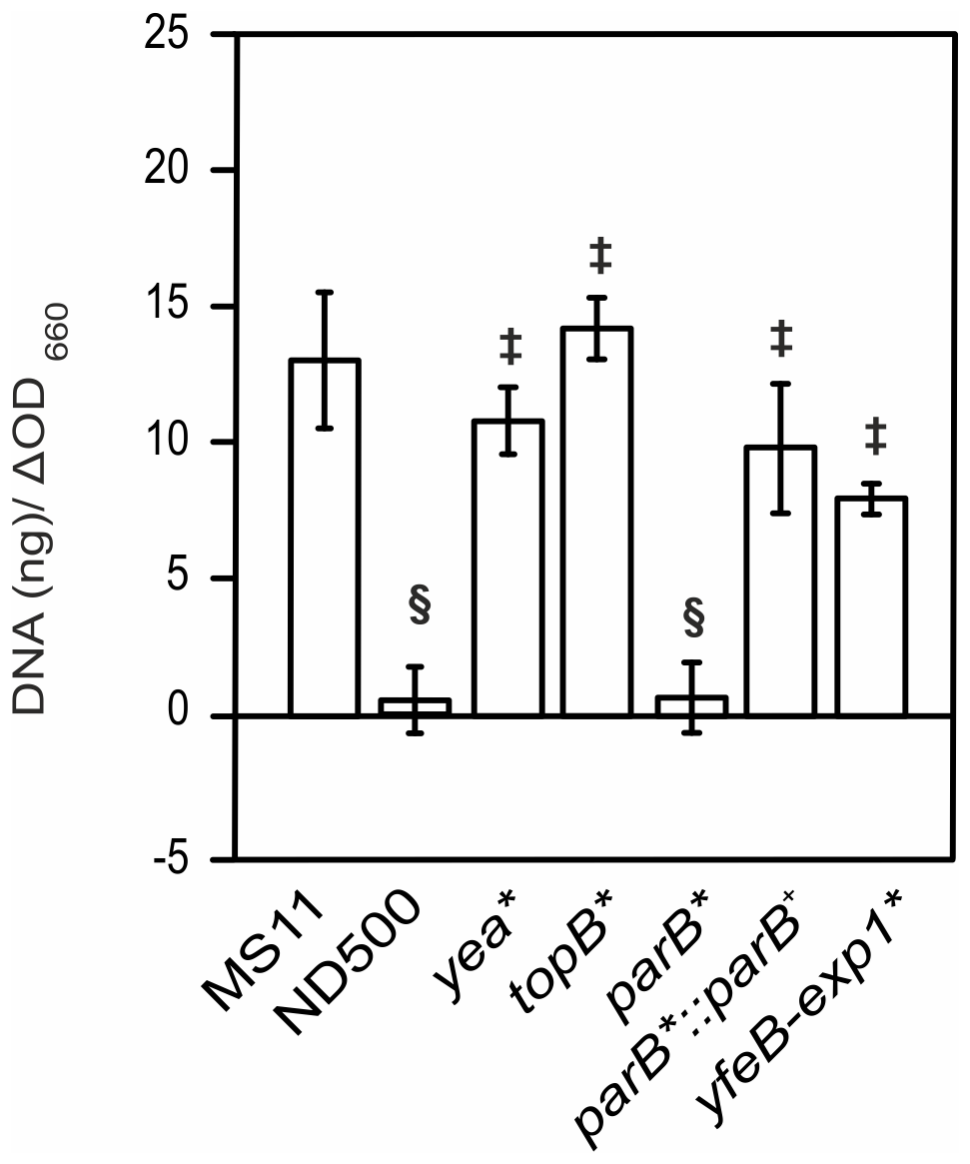
Characterization of the *exp1-parA* region. A) Fluorometric detection of secreted DNA of mutants created in the *exp1-parA* region. Fluorometric detection of secreted DNA was performed as in Figure 2. * indicates a strain in which the respective gene or gene region is disrupted by insertion of an *ermC* containing plasmid via insertion-duplication mutagenesis. parB*::*parB^+^* indicates the strain in which the parB* mutant is complemented by expressing *parB* from the complementation site between the *aspC* and *lctP* genes. § indicates a Student's T-test P-value≤0.05 compared to wild-type; ‡, indicates no statistical difference compared to WT.

**Figure 6:**
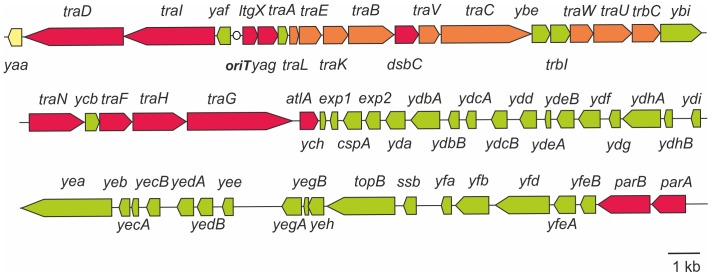
Complete mutational analysis of the genes of the gonoccocal genetic island. Schematic representation of the genetic map of the GGI of *N. gonorrhoeae*. Red and orange indicate genes in which mutations resulted in a decrease in DNA secretion. Green indicates genes in which mutations had no effect on DNA secretion. Yellow indicates *yaa*, of which deletion resulted in an increase of DNA in the medium. For the genes colored in red, secretion could be restored by complementation. For the genes colored in orange no complementation experiments were performed. The dot indicates the origin of transfer (*oriT*).

The region between *exp1* and *parA* contains many open reading frames (ORFs) transcribed in one direction and contains several putative operons. The ORFs mainly encode hypothetical proteins but also several genes with putative functions in DNA processing such as the putative ATP-dependent helicase Yea, the single stranded DNA binding protein SsbB, the topoisomerase TopB, and the partitioning proteins ParA and ParB [8]. This region was not identified in the other GGI-like T4SS. Two mutations were previously generated within this region: *parA*, which strongly affected DNA secretion and *ydbA*, which had no effect on DNA secretion [8] (Figure 1). In a first step, insertion-duplication mutants were created in genes with a putative function: *yea*, *topB*, and *parB* and tested for DNA secretion (Figure 5). The mutations of *yea* and *topB* did not affect DNA secretion demonstrating that these genes were not important for DNA secretion. The strain defective in *parB* showed a strongly reduced DNA secretion, similarly to the previously analyzed *parA* mutant. To confirm the specific effect of *parB* mutation, deletion of *parB* was complemented by *parB* expression from the complementation site between *aspC* and *lctP*. DNA secretion was restored when the *parB* mutant was complemented (Figure 5). This result demonstrates that both partitioning proteins *parA* and *parB* are required for DNA secretion. As mutations in *ydbA*, *yea* and *topB*, which are spread over the *exp1-parA* region, did not affect DNA secretion, a mutant (Δ*exp1-yfeB*) was created in which all genes from *exp1* to *yfeB* were deleted, leaving only *parA* and *parB* of this region. This mutant secreted DNA at similar levels as the WT MS11 strain (Figure 5), demonstrating that genes within the *exp1-yfeB* region are not required for DNA secretion. Thus, mutagenesis of the *parA* [8] and *parB* genes affected DNA secretion, but deletion of the *exp1-yfeB* region did not affect DNA secretion. Previous synteny analysis of this region revealed that the genes encoded in these operons are often found at the borders of large genetic islands, like the PAGI-3(SG), PAGI-2(C) and the *clc*-like genetic islands found in *Pseudomonas aeruginosa* and other organisms [14]. Homologs of the *parA* and *parB* genes were not identified in other GGI-like T4SSs, suggesting that their role in DNA transport might be specific for the *N. gonorrhoeae* T4SS and the PAGI-3(SG), PAGI-2(C) and the *clc*-like genetic islands. ParA and ParB are encoded on many low copy number plasmids and bacterial chromosomes and are involved in plasmid or chromosome partitioning [38]. That suggests that ParA and ParB may be involved in relaxosome formation and its recruitment to the secretion apparatus as it has been shown for *A. tumefaciens* VirC1/VirC2 [39]. Comparison of the flanking regions of GGI-like T4SSs did not reveal any clear conservation of the genes found in the *exp1*-*yfeB* region. Thus our and previous studies (See Figure 6) show that genes that are important for DNA secretion are encoded in the region between *yaa* and *atlA* and include the *parA* and *parB* genes Non of the genes within the *exp1-yfeB* region is important for DNA secretion (See Figure 6). Synteny analysis of all the genes encoded within the *exp1-yfeB* region revealed that no synteny was detected for most of the genes in this region, but that the genes encoding the helicase Yea, the Ydg/YdhA methylase and the conserved hypothetical proteins YedA and YedB show synteny. The order and the distance between the genes differ, but they are often found in the same region. These putative DNA processing proteins are not important for DNA secretion, and their function and the reason for the observed synteny remains unclear. These proteins might be involved in DNA stability of either the integrated, or a putative circular form of the GGI [9], [24].

### The T4SS of the GGI is encoded by 4 operons

**Figure 7:**
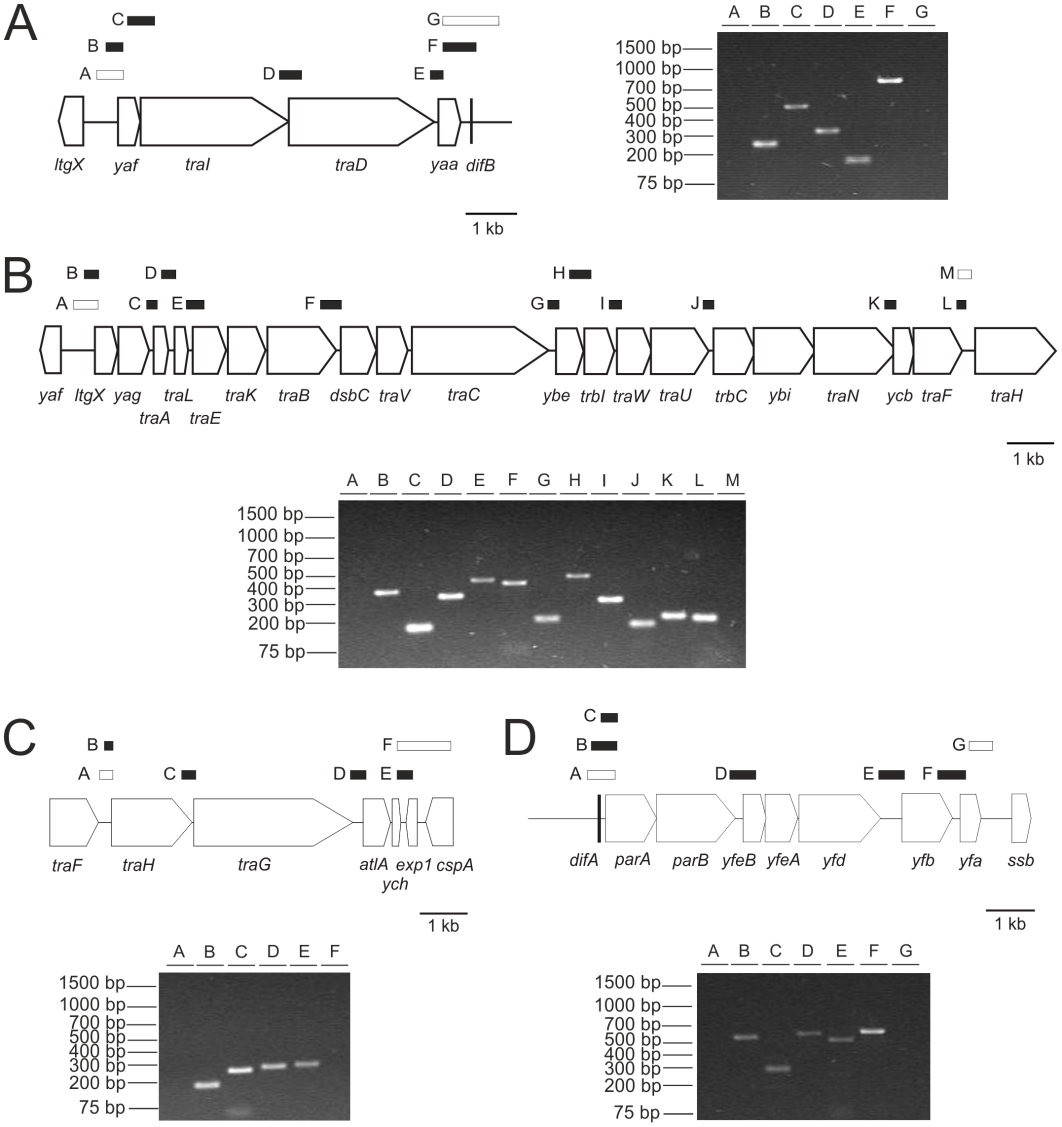
Transcriptional analysis of the genes encoded within the Gonococcal Genetic Island. PCR with different primer combinations on cDNA generated by reverse transcription of RNA isolated from strain MS11 was used to identify operons within the GGI. The results of the transcriptional analysis using different primer pairs within A) the *yaf-yaa*, B), the *ltgX-traF*, C) the *traH-exp1* and D) the *parA-yfa* regions are depicted. The right (A) or lower (B, C, D) part of the figure shows the agarose gels on which the PCR products, obtained with the different primer combinations, were loaded. The left (A) or upper (B, C, D) part of the figure shows a representation of the genetic structure of the operon. Genes are indicated by arrows and the expected PCR products by boxes over the genes. Primer combinations for which a PCR product was obtained are indicated by black boxes and primer combinations for which no PCR product was obtained are indicated by white boxes. The primer combinations used are described in Table S3.

The genetic organization of the genes defined as essential for DNA secretion, and the small intergenic regions suggested that they would be transcribed as several polycistronic messages. We set out to map the operon structure within the regions containing the genes involved in DNA secretion using RT-PCR. cDNA was synthesized from *N. gonorrhoeae* strain MS11, and then the cDNA and specific primers were used to amplify the different intergenic regions. Since it is very likely that gene pairs which are oriented in the same direction and are very close together form an operon, transcription was only analyzed for gene pairs with an intergenic region of ≥10 nucleotides. Successful amplification by these primer pairs was confirmed on chromosomal DNA. No amplification products were detected in control reactions in the absence of reverse transcriptase. This transcriptional mapping approach revealed the presence of at least four different transcripts. The first transcipt includes the region from *yaf* to *yaa* which encodes the components involved in targeting the substrate to the transport complex (Figure 7A). The *ltgX-ych* region encodes the components of the transport complex. The gene order is highly conserved in the GGI-like T4SSs. The second (*ltgX-traF*) and the third (*traH-ych*) transcripts encode the transport complex of the T4SS (Figure 7B and 7C). In the GGI-like T4SSs of *P. mirabilis* and *S. enterica*, large insertions are found between *traN* and *traF*, suggesting that in other GGI-like T4SSs the third transcript might also include the *traF* gene. The fourth transcipt includes *parA*, *parB*, *yfeB*, *yfeA* and *ydf* (Figure 7D) [8], [10], [24]. Thus, the genes required for DNA secretion are encoded on four different transcripts. We have previously identified a fifth transcript within the GGI that includes the genes from *ssbB* to *yegA* [14]. Remarkably, the genes encoded between *yfeB* and *exp1* could be deleted without any effect on DNA secretion. The functions of the genes in this region are not known, but they might encode previously unidentified protein substrates of the T4SS, or other factors that have caused the GGI to be maintained in 80% of the gonococcal strains [11].

## Supporting Information

Table S1 **Strains used in this study.**
(DOCX)

Table S2 **Plasmids and constructs used in this study.** PCR products were created on chromosomal DNA of *N. gonorrhoeae* MS11 unless indicated else.
(DOCX)

Table S3 **PCR primers used in this study.** Primers combinations used for transcriptional mapping are described by the operon they were used to map, a letter which corresponds to the indication in Figure 3 and the indication F or R.
(DOCX)
