# Supplementary material for: Functional Analysis of the Gonococcal Genetic Island of Neisseria gonorrhoeae
Source: PLoS One. 2014 Oct 23;9(10):e109613. doi: 10.1371/journal.pone.0109613 (PMC4207684; doi:10.1371/journal.pone.0109613)
Supplement: Table S1 — Strains used in this study. (DOCX) [file pone.0109613.s001.docx]

**Table S1: Strains used in this stud**y.

| **Strain** | **Genotype** | **source or references** |
| --- | --- | --- |
| *E.coli* DH5α | F^-^ endA1 glnV44 thi-1 recA1 relA1 gyrA96 deoR nupG Φ80d*lacZ*ΔM15 Δ(*lacZYA-argF*)U169, hsdR17(r_K_^-^ m_K_^+^), λ– | Invitrogen |
| MS11 | Laboratory strain of *N. gonorrhoeae* | [1] |
| ND500 | MS11ΔGGI | [2] |
| HH522 | MS11 *exp1*::mTn*CmPhoA* constructed by transformation with pCBB-1 | [2] |
| EP001 | MS11 transformed with pEP007, polar insertion of pIDN3 in *yea,* ErmC | This study |
| EP002 | MS11 transformed with pEP008, polar insertion of pIDN3 in *topB*, ErmC | This study |
| EP003 | MS11 transformed with pEP010, nonpolar insertion of pIDN3 in *traK* | This study |
| EP005 | MS11 transformed with pEP015_2, nonpolar replacement of *traB* by ErmC | This study |
| EP008 | MS11 transformed with pEP025, nonpolar insertion of pIDN3 in *traE*, ErmC | This study |
| EP009 | MS11 transformed with pEP026, nonpolar insertion of pIDN3 in *traW*, ErmC | This study |
| EP010 | MS11 transformed with pEP027, nonpolar insertion in *traU*, ErmC | This study |
| EP011 | MS11 transformed with pEP028, nonpolar insertion of pIDN3 in *trbC*, ErmC | This study |
| EP012 | MS11 transformed with pEP036*,* nonpolar replacement of *traL* by ErmC cassette | This study |
| EP013 | MS11 transformed with pEP037, nonpolar insertion of pIDN3 in *parB*, ErmC | This study |
| EP016 | HH522 transformed with pEP022, ∆ *ych-yfeB*, ErmC | This study |
| EP017 | MS11 transformed with pHH22, in frame deletion of *traA* | This study |
| EP018 | MS11 transformed with pEP020, in frame deletion of *trbI* | This study |
| EP022 | MS11 transformed with pSH003, nonpolar replacement *traV* by ErmC | This study |
| EP046 | EP013 transformed with pEP056, *parB* complementation, Cm | This study |
| EP050 | MS11 transformed with pEP050, in frame deletion of *ybe* | This study |
| JB002 | MS11 transformed with pJB002, nonpolar replacement *traC* by ErmC | This study |
| SI10 | MS11 transformed with pSI10, deletion of *yag* | This study |
| SI11 | MS11 transformed with pSI11, deletion of *ycb* | This study |
| SI12 | MS11 transformed with pSI12, deletion of *ybi* | This study |
| JD1614 | MS11 transformed with pJD1188, mTn*CmNS* insertion in *ych* | This study |
| PK153 | SI10 transformed with pPK1007, *yag* complementation | This study |
| TB001 | MS11 background, *yaf* marker-less in-frame deletion by double-homologous recombination | This study |
| TB002 | MS11 transformed with pTB009, nonpolar insertion of pIDN3 in *yaa*, Erm | This study |
| KL505 | MS11 with 4 bp insertion in *yaa* resulting in frameshift | This study |
| TB003 | Restoration of the *yaa*-locus in KL505 by double-homologous recombination of wt *yaa* DNA | This study |

1. Swanson J, Kraus SJ, Gotschlich EC (1971) Studies on gonococcus infection. I. Pili and zones of adhesion: their relation to gonococcal growth patterns. J Exp Med 134: 886-906.

2. Hamilton HL, Dominguez NM, Schwartz KJ, Hackett KT, Dillard JP (2005) Neisseria gonorrhoeae secretes chromosomal DNA via a novel type IV secretion system. Mol Microbiol 55: 1704-1721.
