# Supplementary material for: Functional Analysis of the Gonococcal Genetic Island of Neisseria gonorrhoeae
Source: PLoS One. 2014 Oct 23;9(10):e109613. doi: 10.1371/journal.pone.0109613 (PMC4207684; doi:10.1371/journal.pone.0109613)
Supplement: Table S2 — Plasmids and constructs used in this study. PCR products were created on chromosomal DNA of N. gonorrhoeae MS11 unless indicated else. (DOCX) [file pone.0109613.s002.docx]

**Table S2: Plasmids and constructs used in this study**. PCR products were created on chromosomal DNA of *N. gonorrhoeae* MS11 unless indicated else.

| **plasmid** | **properties** | **source or references** |
| --- | --- | --- |
| pIDN1 | IDM vector (Erm^R^) | [1] |
| pIDN2 | IDM vector (Erm^R^) | [1] |
| pIDN3 | IDM vector (Erm^R^) | [1] |
| pKH35 | Complementation vector (Cm^R^) | [2] |
| pKH37 | Complementation vector (Cm^R^) | [3] |
| pKS68 | Plasmid to construct *ycb* deletion | This study |
| pKS78 | Plasmid to construct *ybi* deletion | This study |
| pKS96 | Plasmid to construct *yag* deletion | This study |
| pKL8 | Plasmid to construct *yaa* frame-shift mutation. | This study |
| pJD1103 | Plasmid to construct *ych* insertion mutation | [4] |
| pJD1188 | Plasmid to construct *ych* insertion. Shuttle mutagenesis of pJD1103 with mTn*CmNS* generating pJD1188 | This study |
| pSH001 | Cloning vector (Erm^R^), created by ligation of PCR products created using pIDN1 as template with primers ForwardDUS and ReverseDUS, and primers ForwardErmC and ReverseErmC, digested with EcoRI and HindIII | This study |
| pSH002 | Plasmid to construct *traV* deletion. Contains PCR product of a *dsbC* fragment created with primers GGI-100F and GGI-101R cloned into HindIII and XhoI sites of pSH001 | This study |
| pSH003 | Plasmid to construct *traV* deletion. Contains *traC* fragment created with primers GGI-102F and GGI-103R cloned in EcoRI and PstI sites of pSH002 | This study |
| pEP007 | Plasmid to construct *yea* deletion via insertion-duplication mutagenesis. Contains PCR product of a *yea* fragment created with primers GGI-25F and GGI-26R cloned in SacI and KpnI sites of pIDN3 | This study |
| pEP008 | Plasmid to construct *topB* deletion via insertion-duplication mutagenesis. Contains PCR product of a *topB* fragment created with primers GGI-27F and GGI-28R cloned in BamHI and KpnI sites of pIDN3 | This study |
| pEP010 | Plasmid to construct *traK* deletion via insertion-duplication mutagenesis. Contains PCR product of a *traK* fragment created with primers GGI-21F and GGI-22R cloned in BamHI and KpnI sites of pIDN3 | This study |
| pEP015_1 | Plasmid to construct *traB* replacement via insertion-duplication mutagenesis. Contains PCR product of a *traB* fragment created with primers GGI-87F and GGI-88R cloned in HindIII and KpnI sites of pSH001 | This study |
| pEP015_2 | Plasmid to construct *traB* replacement via insertion-duplication mutagenesis. Contains PCR product of a *traB* fragment created with primers GGI-89F and GGI-90R cloned in EcoRI and SacI sites of pEP015_1 | This study |
| pEP016 | Plasmid to construct *trbI* deletion via insertion-duplication mutagenesis. Contains PCR product of a *ybe* fragment created with primers GGI-91F and GGI-92R cloned in SacI and BamHI sites of pIDN1 | This study |
| pEP020 | Plasmid to construct *trbI* deletion via insertion-duplication mutagenesis. Contains PCR product of a *traW* fragment created with primers GGI-93F and GGI-94R cloned in EcoRI and KpnI sites of pEP016 | This study |
| pEP021 | Plasmid to construct the *exp1-yfeB* deletion. Contains PCR product of *atlA-exp1* fragment created with primers GGI-105F and GGI-106R cloned in SalI and ApaI sites of pIDN2 | This study |
| pEP022 | Plasmid to construct the *exp1-yfeB* deletion. Contains PCR product of *parA-parB* fragment created with primers GGI-107F and GGI-108R cloned in EcoRI and NotI sites of pEP021 | This study |
| pEP025 | Plasmid to construct *traE* deletion via insertion-duplication mutagenesis. Contains PCR product of a *traE* fragment created with primers GGI-132F and GGI-133R cloned in BamHI and SacI sites of pIDN3 | This study |
| pEP026 | Plasmid to construct *traW* deletion via insertion-duplication mutagenesis. Contains PCR product of a *traW* fragment created with primers GGI-134F and GGI-135R cloned in XhoI and HindIII sites of pIDN3 | This study |
| pEP027 | Plasmid to construct *traU* deletion via insertion-duplication mutagenesis. Contains PCR product of a *traU* fragment created with primers GGI-140F and GGI-141R cloned in XhoI and HindIII sites of pIDN3 | This study |
| pEP028 | Plasmid to construct *trbC* deletion via insertion-duplication mutagenesis. Contains PCR product of a *trbC* fragment created with primers GGI-136F and GGI-137R cloned in XhoI and HindIII sites of pIDN3 | This study |
| pEP035 | Plasmid to construct *traL* deletion via insertion-duplication mutagenesis. Contains PCR product of a *traA* fragment created with primers GGI-161F and GGI-162R cloned in HindIII and XhoI sites of pSH001 | This study |
| pEP036 | Plasmid to construct *traL* deletion via insertion-duplication mutagenesis. Contains PCR product of a *traE* fragment created with primers GGI-163F and GGI-164R cloned in BamHI and SacI sites of pEP035 | This study |
| pEP037 | Plasmid to construct *parB* deletion via insertion-duplication mutagenesis. Contains PCR product of a *parB* fragment created with primers GGI-165F and GGI-166R cloned in BamHI and SacI sites of pIDN3 | This study |
| pEP050 | Plasmid to construct *ybe* deletion via insertion-duplication mutagenesis. Contains PCR product of a *trbI* fragment created with primers GGI-216F and GGI-217R cloned in KpnI and HindIII sites of pEP049 | This study |
| pEP056 | Plasmid to construct *parB* complementation *in trans*. Contains PCR product of a *parB* created with primers GGI-224F and GGI-225R cloned in SalI and SacI sites of pKH35 | This study |
| pJB001 | Plasmid to construct *traC* deletion via insertion-duplication mutagenesis. Contains PCR product of a *traV* fragment created with primers GGI-65F and GGI-66R cloned in BamHI and SacI sites of pIDN3 | This study |
| pJB002 | Plasmid to construct *traC* deletion via insertion-duplication mutagenesis. Contains PCR product of a *ybe* fragment created with primers GGI-67F and GGI-68R cloned in XhoI and EcoRI sites of pJB001 | This study |
| pHH22 | Plasmid to construct *traA* deletion via double crossover recombination | This study |
| pKL9 | Plasmid to construct *yaa* frame-shift mutation. Contains 4bp insertion in *yaa* coding sequence. | This study |
| pSI10 | Plasmid to construct *yag* deletion. Contains PCR product created with primers yag3’F and yag5’R | This study |
| pSI11 | Plasmid to construct *ycb* deletion. Contains PCR product created with primers ycbBsaF and ycbBsaR | This study |
| pSI12 | Plasmid to construct *ybi* deletion. Derivative of pKS78 by digestion with PstI | This study |
| pPK1007 | Plasmid to construct *yag* complementation *in trans*. Contains PCR product of *yag* created with primers yagRBSF and 48R cloned in HindIII and SpeI sites of pKH37 | This study |
| pTB009 | Plasmid to construct *yaa* deletion via insertion-duplication mutagenesis. Contains PCR product of a *yaa* fragment created with primers 820F-GGI and 821R-GGI cloned in NotI and SacI sites of pIDN3 | This study |

1. Hamilton HL, Schwartz KJ, Dillard JP (2001) Insertion-duplication mutagenesis of neisseria: use in characterization of DNA transfer genes in the gonococcal genetic island. J Bacteriol 183: 4718-4726.

2. Hamilton HL, Dominguez NM, Schwartz KJ, Hackett KT, Dillard JP (2005) *Neisseria gonorrhoeae* secretes chromosomal DNA via a novel type IV secretion system. Mol Microbiol 55: 1704-1721.

3. Kohler PL, Hamilton HL, Cloud-Hansen K, Dillard JP (2007) AtlA functions as a peptidoglycan lytic transglycosylase in the *Neisseria gonorrhoeae* type IV secretion system. J Bacteriol 189: 5421-5428.

4. Dillard JP, Seifert HS (1997) A peptidoglycan hydrolase similar to bacteriophage endolysins acts as an autolysin in *Neisseria gonorrhoeae*. Mol Microbiol 25: 893-901.
