# Supplementary material for: Functional Analysis of the Gonococcal Genetic Island of Neisseria gonorrhoeae
Source: PLoS One. 2014 Oct 23;9(10):e109613. doi: 10.1371/journal.pone.0109613 (PMC4207684; doi:10.1371/journal.pone.0109613)
Supplement: Table S3 — PCR primers used in this study. Primers combinations used for transcriptional mapping are described by the operon they were used to map, a letter which corresponds to the indication in Figure 3 and the indication F or R. (DOCX) [file pone.0109613.s003.docx]

**Table S3:** **PCR primers used in this study.** Primers combinations used for transcriptional mapping are described by the operon they were used to map, a letter which corresponds to the indication in Figure 3 and the indication F or R.

| **Oligo name** | | **5’- 3’** | | **description** |
| --- | --- | --- | --- | --- |
|  | | **Primers used to create plasmids and strains** | |  |
| GGI-21F | | GGGGGTACCACGGAACTTGAGCAGAATCG | | forward *traK* with KpnI site |
| GGI-22R | | AAAGGATCCGTAGTGCCACGCATCATAGA | | reverse *traK* with BamHI site |
| GGI-25F | | AAAGAGCTCAGGTCCAGAGCGGTTCCTTA | | forward *yea* with SacI site |
| GGI-26R | | AAAGGTACCCACCGCACCTTGGTATTGAG | | reverse *yea* with KpnI site |
| GGI-27F | | CGCGGATCCCTGGCCTTGGTCGGAATAAT | | forward *topB* with BamHI site |
| GGI-28R | | AAAGGTACCCTGGCGTAATACTGACGGAT | | reverse *topB* with KpnI site |
| GGI-43F | | GGGGTCATGAGGTTCATAATGAATATCAAACG | | forward *traA* with BspHI site |
| GGI-65F | | GTGGGATCCGGAGCGGGAGCAGAATGATG | | forward *traV* with BamHI site |
| GGI-66R | | CCGGAGCTCTGACAGGACGCCCATTACATAC | | reverse *traV* with SacI site |
| GGI-67F | | GTACTCGAGCCGCTAATTAGGCAGAGGATAGG | | forward *ybe* with EcoRI site |
| GGI-68R | | TCCGAATTCCTTTACCCTCTTCGGCCATC | | reverse *ybe* with XhoI site |
| GGI-87F | | GCGGAAGCTTGGAGGTTGAGATGAGGGTGAAAG | | forward *traB* with HindIII site |
| GGI-88R | | CTGCGGTACCGATACCGCTAATTGCAGGCG | | reverse *traB* with KpnI site |
| GGI-89F | | CGCGAATTCTCAGAACGCGCTTACATCAG | | forward *traB* with EcoRI site |
| GGI-90R | | CGCGAGCTCCAGTACGACATCGACTTGAC | | reverse *traB* with SacI site |
| GGI-91F | | GGCGAGCTCGGAACAGGCGAAGAATCAAG | | forward *ybe* with SacI site |
| GGI-92R | | CGCGGATCCCTCAAACATTAACGATCCATTTC | | reverse *ybe* with BamHI site |
| GGI-93F | | CGCGAATTCCCATTCAGGGCAAATTATTGGTAAG | | forward *traW* with EcoRI site |
| GGI-94R | | GCGGGTACCGCTGCTTCCAAAGGTTTGAC | | reverse *traW* with KpnI site |
| GGI-100F | | GCGAAGCTTTACCGGCGAAACCAAATAG | | forward *dsbC* with HindIII site |
| GGI-101R | | GGGCTCGAGGCCGCAGTAATCGATAAG | | reverse *dsbC* with XhoI site |
| GGI-102F | | CGCGAATTCCCAGGAAACATCGGGTATTCCCC | | forward *traC* with EcoRI site |
| GGI-103R | | GGGCTGCAGAGGATACAGCGCAGATTTCG | | reverse *traC* with PstI site |
| GGI-105F | | ATATGTCGACTCCGCCCAAAGGAATAACCC | | forward *atlA-exp1* with SalI site |
| GGI-106R | | TGCGGGCCCTTCAGCACAGGCGAAGTGAC | | reverse *atlA-exp1* with ApaI site |
| GGI-107F | | TATGAATTCTCATCCGCTCGACAATTTCC | | forward *parA-parB* with EcoRI |
| GGI-108R | | GCGGCGGCCGCCGGCGTATTTGGTGCGGATG | | reverse *parA-parB* with NotI site |
| GGI-132F | | AGCCTCGAGAATCCGGCGGTCAATGACTG | | forward *traE* with XhoI site |
| GGI-133R | | GCGAAGCTTGCGGGTTCTGCTTTAGTTAC | | reverse *traE* with HindIII |
| GGI-134F | | CGCCTCGAGAACCGGAATTTCCTCATGGG | | forward *traW* with XhoI site |
| GGI-135R | | GCGAAGCTTTTGGCCAGTTCGATTTGGTC | | reverse *traW* with HindIII site |
| GGI-136F | | CGGCTCGAGCCCAAATGTGGGTGACAATC | | forward *trbC* with XhoI site |
| GGI-137R | | GCGAAGCTTTACAGCGCAACCGTCTAAAC | | reverse *trbC* with HindIII site |
| GGI-140F | | GCGCTCGAGCAAGAGGATAACGGCTCTTC | | forward *traU* with XhoI site |
| GGI-141R | | GCGAAGCTTGGCCGCTTTAACACAGTCAG | | reverse *traU* with HindIII site |
| GGI-161F | | CGTAAGCTTGCTAGTGCGGTTGCATTGGCATTG | | forward *traA* with HindIII site |
| GGI-162R | | GCTCTCGAGCCTAATGTTGCGAGCCAACA | | reverse *traA* with XhoI site |
| GGI-163F | | GCGCGGATCCCGGGAGTTTCTAAATTGAAG | | forward *traE* with BamHI site |
| GGI-164R | | CGCGAGCTCGTTGCTTCTGTCGCGCTAAA | | reverse *traE* with SacI site |
| GGI-165F | | TCTGAGCTCGCTTTGGCTGGCACAAATTC | | forward *parB* with SacI site |
| GGI-166R | | TACGGATCCCCCTACTCTGACGAAACTAC | | reverse *parB* with BamHI site |
| GGI-190F | | TCAGGGCCCTTTCCTGGCTAATGGCTCTC | | forward *traD* with ApaI site |
| GGI-191R | | GCGGTCGACGGGAAGCTTACCCAAAGGTCAAGCATTCG | | reverse *traD* with SalI site |
| GGI-214F | | CTAGAGCTCGGCACGGTCGATCATGTAAG | | forward *traC* with SacI site |
| GGI-215R | | CGCGGATCCTCTGCCTAATTAGCGGGTTGC | | reverse *traC* with BamHI site |
| GGI-216F | | GCGAAGCTTGGTAAAGGCACCCCGGTTAC | | forward *trbI* with HindIII site |
| GGI-217R | | CGCGGTACCTACTAGCGATGCGACTGAAG | | reverse *trbI* with KpnI site |
| GGI-224F | | CTCGTCGACCAGAAGAACCTGCGTGGAAG | | forward parB with SalI site |
| GGI-225R | | GTCGAGCTCTTACTCCTCACTCTTAGCTCCCGTTTTTCTC | | reverse *parB* with SacI site |
| ycbBsaF | | TCGGGTCTCAATAACAGAAAGAACGGTGGTAAA | | forward *ycb* with BsaI site |
| ycbBsaR | | AGCGGTCTCATTATTGATATTCATAATAGTTCTTC | | reverse *ycb* with BsaI site |
| yag3’F | | GTAGGTCTCTTGTAAAGAATGATCGGACGCA | | forward *yag* with BsaI site |
| yag5’R | | GGCGGTCTCATACATTGCGGAGGCTACGAAC | | reverse *yag* with BsaI site |
| yagRBSF | | GACACTAGTAGGAGGTCCGCAATGATTCTGACAGCC | | forward *yag* with HindIII site |
| 48R | | CCATTGATGCTTGCCAGACT | | reverse *yag* with SpeI site |
| ForwardDUS | | CCGAAGCTTGAGCTTGCCGTCTGAA | | forward DUS |
| ReverseDUS | | TATGGAATTCCTGCAGCCCGGGGATCC | | reverse DUS |
| ForwardErm | | TACTGCCGGCCGCTCTAGAACTA | | forward ErmC |
| ReverseErm | | TCGGAATTCGCTGCATGCCGTCT | | reverse ErmC |
| dif-F | | ACTGGATCCCTCGAGCTCGTGCGAATGCGGCCAAAATCTATACC | | forward *parA* with BamHI site |
| dif-R | | CCGCGGTTAAATCTGGGTATATTTAACATAATATACATTATGCGAACTATCGGAA | | reverse *parA* with SacII site |
| 41 | | CCAGGCGATTTCGGCATGTG | | 5’ fragment for *yaf*-deletion |
| 803R-GGI | | GAGACTCGAGGAGATATTTCTTAAGATATTTAGCTTGCATGAAAATC | | amplification of *yaa* after restoration |
| 812R-GGI | | CAGCGAGAATTCAAATCTTTGCTTAAATCATCATG | | 5’ fragment for *yaf*-deletion with EcoRI-site |
| 808R-GGI | | GCCGTCTGAAGAAAGCGCTCTCGGTTAATG | | 3’ fragment for *yaf*-deletion |
| 813F-GGI | | AGCTAGGAATTCGGGAAGATCTGTTTCATTCATTTTG | | 3’ fragment for *yaf*-deletion with EcoRI-site |
| 820F-GGI | | CATGACTGAGCGGCCGCGGCCATGATGCGTT-  TCCAGGAAAAGAGGTTGACGATAC | | forward *yaa* with NotI-site |
| 821R-GGI | | GCTGCTGAGCTCACGGTGATAAATGCCTGCACTC | | reverse *yaa* with SacI-site |
| 832F-GGI | | ATGCCGTCTGAAAAAACAGGAAGTGGCCGGGCTGCCTC | | restoration-product of *yaa* in KL505 |
| 833R-GGI | | ATGCCGTCTGAAGAGAAGATAGGCAGCATAGG | | restoration-product of *yaa* in KL505 |
|  | | **Primers used for operon mapping** | |  |
| 914 | | 5’-ACCGGCTACACGTACAACTG-3’ | | *yaf-yaa*-F-For |
| 915 | | 5’-GCTGGGACATATTGGAATGG-3’ | | *parA-yfa*-B-Rev |
| 926 | | 5’-CAGACGCTCCATTCTCGAAG-3’ | | *yaf-yaa*-G-For |
| 1048 | | 5’-GATTGCGGATGTTTTTCTCTTTATAAATC-3’ | | *parA-yfa*-C-Rev |
| 1050 | | 5’-GCAATGGCGGAGGAATTCAC-3’ | | *parA-yfa*-A-Rev |
| 418F-GGI | | 5’-AAGCATGATGAGGGCTATGG-3’ | | *traH-exp1*-C-For |
| 476F-GGI | | 5’-TGGTCGGACGGAACACAGAG-3’ | | *ltgX-traF*-H-For |
| 477R-GGI | | 5’-CGACCTGCGTACCAATAGGG-3’ | | *ltgX-traF*-H-Rev |
| 478F-GGI | | 5’-GGCCTGCTGCAATAGTGATG-3’ | | *ltgX-traF*-C-For |
| 479R-GGI | | 5’-ACCGCACTAGCGGACTTTAC-3’ | | *ltgX-traF*-C-Rev |
| 480F-GGI | | 5’-CAGCTTGGACAGTCGATATG-3’ | | *ltgX-traF*-I-For |
| 481R-GGI | | 5’-TTGGCCGCTGTCTTGTTTAG-3’ | | *ltgX-traF*-I-Rev |
| 482F-GGI | | 5’-AGACAGCGGCAAAGCATTTC-3’ | | *ltgX-traF*-J-For |
| 483R-GGI | | 5’-CCGGCTACGATTACATTGCG-3’ | | *ltgX-traF*-J-Rev |
| 484F-GGI | | 5’-AGGGAGGCATCCGTTAATGG-3’ | | *ltgX-traF*-K-For |
| 485R-GGI | | 5’-GATGGAAGTCCTTGCTAGAG-3’ | | *ltgX-traF*-K-Rev |
| 486F-GGI | | 5’-ATGTCCGGCTTCGGTATAGG-3’ | | *ltgX-traF*-E-For |
| 487F-GGI | | 5’-ATCAAGCAGCACGCATTTGG-3’ | | *traH-exp1*-D-For |
| 488R-GGI | | 5’-CGCACTTGCGGATTATGAAC-3’ | | *traH-exp1*-D-Rev |
| 489F-GGI | | 5’-TAGCGTATTCCCGCCCTGTC-3’ | | *ltgX-traF*-LM -For |
| 491F-GGI | | 5’-TACAGCCGAGGCCATTGAAG-3’ | | *ltgX-traF*-G-For |
| 492R-GGI | | 5’-CAAGGCTGCCCAATGAAACC-3’ | | *ltgX-traF*-G-Rev |
| 493F-GGI | | 5’-TAAGGTCTTCCCGGTAGTTG-3’ | | *ltgX-traF*-F-For |
| 494R-GGI | | 5’-ATGGCAGTCGGGAATAACTC-3’ | | *ltgX-traF*-F-Rev |
| 495F-GGI | | 5’-CCGCTAGTGCGGTTGCATTG-3’ | | *ltgX-traF*-D-For |
| 496R-GGI | | 5’-GATACCGGCACATGATAATCTC-3’ | | *ltgX-traF*-D-Rev |
| 706R-GGI | | 5’-GCCGTATGTCGAGAAAGAAG -3’ | | *parA-yfa*-G-Rev |
| 707R-GGI | | 5’-GCAGCATAGGGAGCCATTTC-3’ | | *ltgX-traF*-E-Rev |
| 710F-GGI | | 5’-TGTGTCAACACCGAACTACC-3’ | | *yaf-yaa*-E-For |
| 711R-GGI | | 5’-AACGCATTTACGGAGGGAAG-3’ | | *yaf-yaa*-E-Rev |
| 712F-GGI | | 5’-TTCCAGATAACCGCTAGCAC-3’ | | *yaf-yaa*-AB-For |
| 713F-GGI | | 5’-CGGCCACTGGAAGAAACAAC-3’ | | *parA-yfa*-F-For |
| 714R-GGI | | 5’-GAGACCAGGGCTATCAAGAG-3’ | | *parA-yfa*-F-Rev |
| 716R-GGI | | 5’-GAAAGCGCTCTCGGTTAATG-3’ | | *ltgX-traF*-AB-Rev |
| 717R-GGI | | 5’-AAACGGGAGCTAAGAGTGAG-3’ | | *parA-yfa*-D-Rev |
| 718F-GGI | | 5’-TTGGGCAAGGCTATAATCGG-3’ | | *parA-yfa*-D-For |
| 721R-GGI | | 5‘-TCTGTGACAATTCTAATTAAAATAAC-3‘ | | *yaf-yaa*-B-Rev |
| 722F-GGI | | 5’-AGGGAAGGGCATCCTTACTC-3’ | | *yaf-yaa*-C-For |
| 723R-GGI | | 5’-GCCACTGCCGATAGATATTG-3’ | | *yaf-yaa*-C-Rev |
| 724F-GGI | | 5’-GGCAGTAAGGGCATAATAGG-3’ | | *yaf-yaa*-D-For |
| 725R-GGI | | 5’-CATACAGCCAGGTTCAAGAC-3’ | | *yaf-yaa*-D-Rev |
| 728F-GGI | | 5’-ATATCTAGCTAAAATGCCCACGGACAG-3’ | | *parA-yfa*-G-For |
| 730R-GGI | | 5’-TGTTGGCCATGATGCGTTTC-3’ | | *yaf-yaa*-FG-Rev |
| 733F-GGI | | 5’-GAGGTAACGATCTAGTATCC-3’ | | *traH-exp1*-EF-For |
| 734R-GGI | | 5’-TGCTCAAGTAGTGATTTAGG-3’ | | *traH-exp1*-E-Rev |
| 739R-GGI | | 5’-GCCGGTTCAGATATACCAGG-3’ | | *traH-exp1*-F-Rev |
| 743R-GGI | | 5’-ATTGGCTTCCGCTCCCATTG-3’ | | *traH-exp1*-AB-Rev |
| 744F-GGI | | 5’-GCTATAACCGCTTCATGGAG-3’ | | *traH-exp1*-A-Rev |
| 746F-GGI | | 5’-GACAACGCGGATATTTCAGG-3’ | | *traH-exp1*-B-Rev |
| 748R-GGI | | 5’-AATATCCGCGTTGTCAACCG-3’ | | *ltgX-traF*-L-For |
| 750R-GGI | | 5’-GCCGGCTTCGGAAAGATGTG-3’ | | *ltgX-traF*-M-For |
| 774R-GGI | | 5’-GGCAGCATTATACCTTATAAATC-3’ | | *yaf-yaa*-B-Rev |
| 775R-GGI | | 5’-TCAAGGGAAAAAGGGTAAAAG-3’ | | *yaf-yaa*-A-Rev |
| 776R-GGI | | 5’-GCAACAGCAAGAGTGACCAG-3’ | | *traH-exp1*-C-Rev |
| 778F-GGI | | 5’-GCCTTTACCCTTATCGTATTC-3’ | | *ltgX-traF*-B-For |
| 779F-GGI | | 5’-CTTGAACCCTTCCTTTAACC-3’ | | *ltgX-traF*-A-For |
| 784F-GGI | | 5’-AGCCAAAGCAGCACGAGCCATATC-3’ | | *parA-yfa*-E-For |
| 785R-GGI | | 5’-TAACCTATGCCCGCTGCGCTTC-3’ | | *parA-yfa*-E-Rev |
| 786F-GGI | | 5’-ATTGTCGAGCGGATGATTTC-3’ | | *parA-yfa*-ABC-For |
|  | | **Primers used for qPCR** | |  |
| 697 | | GCTTACGGCGTTGCTTATTG | | *secY* |
| 698 | | CCCGCCCTACCATTAAACTG | | *secY* |
| 472 R-GGI | | GATATGCCCGAGTCTGAAGC | | *traD* |
| 473 F-GGI | | CCCAATGCGTCAATAAGAGG | | *traD* |
| 474 R-GGI | | GTCTATCCAACCGGTGACAG | | *traI* |
| 475 F-GGI | | CCCGGTTCTTTAGCTTTCTC | | *traI* |
| 834 F-GGI | | GTTCATGGCTACCCGTACTC | | *ltgx* |
| 835 R-GGI | | GTACATTTGTGCATGGATTCCATAC | | *ltgX* |
| 836 F-GGI | | GAAGAACTATACGGGTCAACG | | *traH* |
| 837 R-GGI | | GAAACTTTGAATACCTACTGTGTATTTG | | *traH* |
| 838 F-GGI | | GCATTCCCAATACATACAGAATAAC | | *parA* |
| 839 R-GGI | | CAGGCATGTAGCAGCAAATG | | *parA* |
|  |  | |  | |
